# Supplementary material for: Non-flammable solvent-free liquid polymer electrolyte for lithium metal batteries
Source: Nat Commun. 2023 Aug 1;14:4617. doi: 10.1038/s41467-023-40394-8 (PMC10394022; doi:10.1038/s41467-023-40394-8)
Supplement: Supplementary file 1 — Supplementary Information [file 41467_2023_40394_MOESM1_ESM.pdf]

Non-flammable solvent-free liquid polymer electrolyte for lithium metal batteries

Guo-Rui Zhu[1], Qin Zhang[1], Qing-Song Liu[1], Qi-Yao Bai[1], Yi-Zhou Quan[1], You Gao[1], Gang Wu[1]*, Yu-Zhong Wang[1]*

[1]The Collaborative Innovation Center for Eco-Friendly and Fire-Safety Polymeric Materials (MoE), National Engineering Laboratory of Eco-Friendly Polymeric Materials (Sichuan), State Key Laboratory of Polymer Materials Engineering, College of Chemistry, Sichuan University, Chengdu 610064, China.

E-mail address: gangwu@scu.edu.cn; yzwang@scu.edu.cn

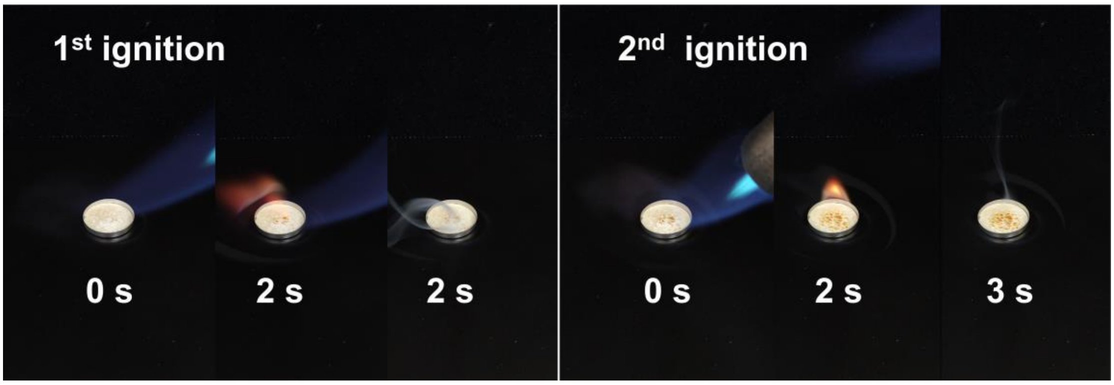

Supplementary Figure 1. Continuous butane flame ignition test of LPE (GF-PPZ-16). The ignition time is 2 s.

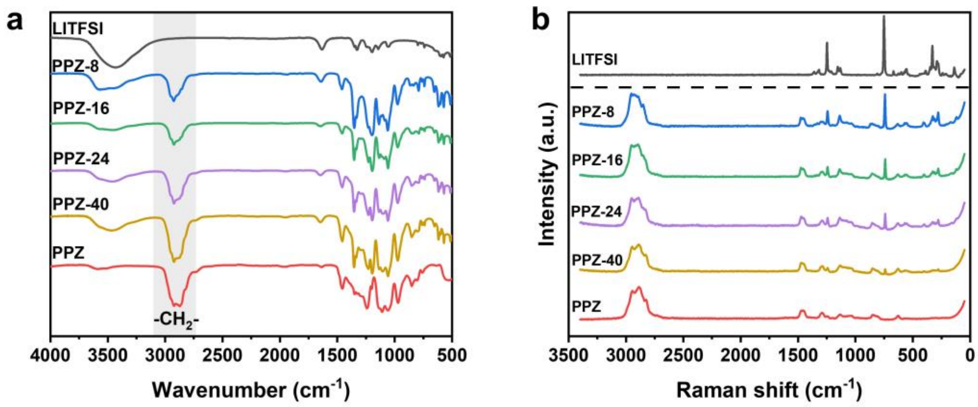

Supplementary Figure 2. Spectral analysis of LPE electrolytes. **a** FTIR spectra; **b** Raman spectra.

The P=N-P (1200 cm^{-1} to 1230 cm^{-1}) absorption twin peak of PPZ produced a significant peak shape change due to the coordination of N atoms and side chains with Li^{+}. The P=N and P-N peaks are shifted to low wavenumber by 2 cm^{-1} and 5 cm^{-1}, respectively. The stretching vibration of C-O-C (1275-1020 cm^{-1}) shifts about 2 cm^{-1} to high wavenumber, while the relative strength decreases with increasing salt content. Similarly, the P-O-C absorption peak (970 cm^{-1}) moved about 4 cm^{-1}. All the changes showed a strong correlation with the regular changes in the lithium salt concentration.

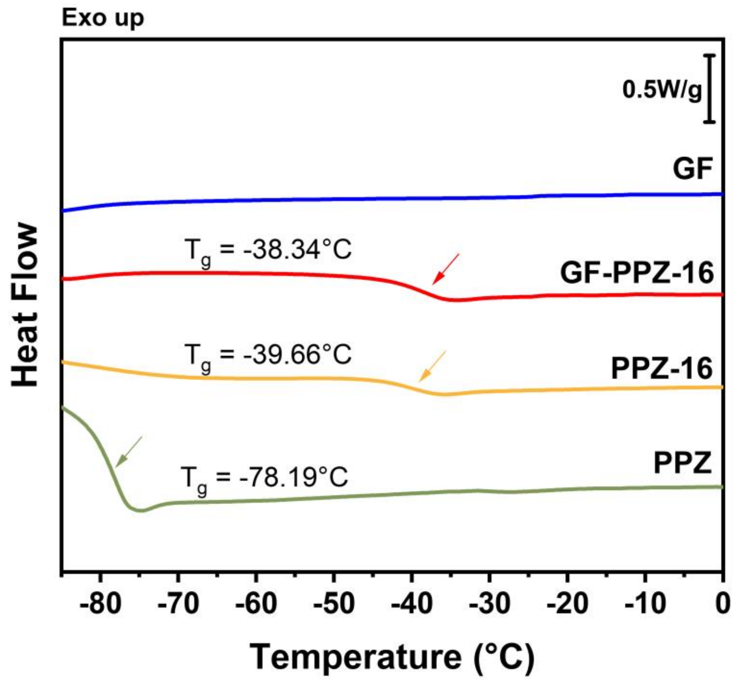

Supplementary Figure 3. DSC curves.

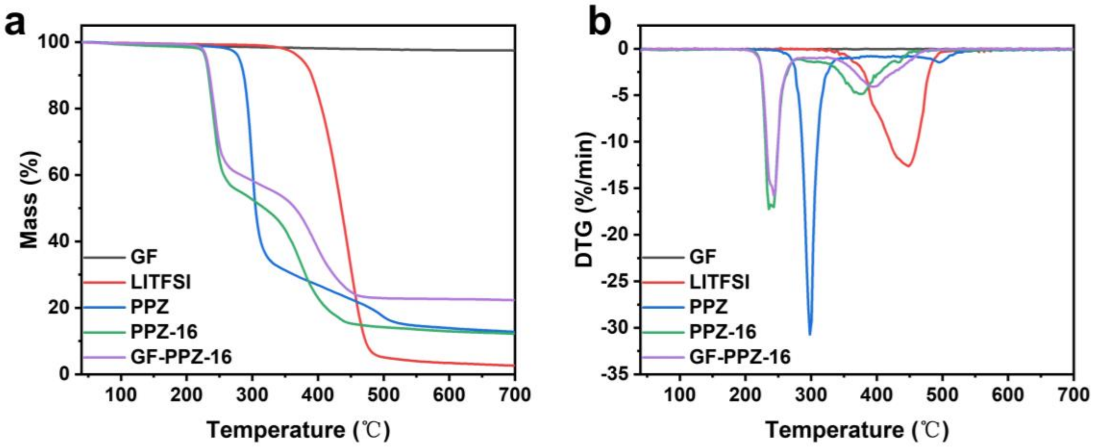

Supplementary Figure 4. Thermal decomposition curves of GF-PPZ-16 electrolyte and its components. **a** TG and **b** DTG curves of different samples.

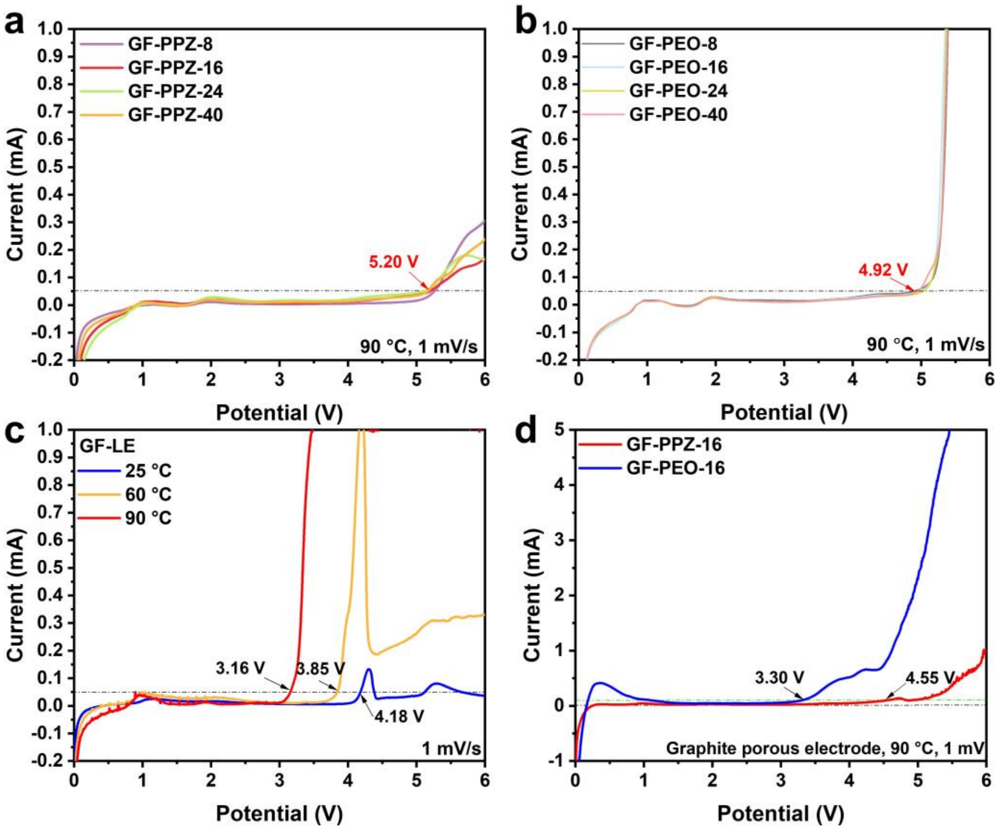

Supplementary Figure 5. Electrochemical windows tests. The LSV curves of **a** PPZ-based LPEs, **b** PEO-based SPEs, and **c** commercial LE with stainless steel flat plate electrodes. **d** The LSV curves of GF-FPPZ-16 and GF-PEO-16 electrolytes with graphite porous electrodes. GF as a separator.

The PPZ-based LPEs exhibited a wide electrochemical window of 5.20 V based on flat plate electrodes at 90°C, which exceeded LE's 3.16 V and PEO-based SPE's 4.92 V. Notably, commercial LE exhibited poor oxidation stability, and the electrochemical window decreased significantly with increasing temperature in Supplementary Figure 5c. Compared with the flat electrode, the porous electrode is closer to the actual situation. As shown in Supplementary Figure 5d, the carbon porous electrode made the electrochemical window visibly lower by accelerating the interfacial reaction kinetics[1], which was due to the catalysis and larger interfacial contact[2, 3, 4]. Despite this, GF-PPZ-16 still exhibited an electrochemical window of up to 4.55V, significantly better than GF-PEO-16's 3.30V.

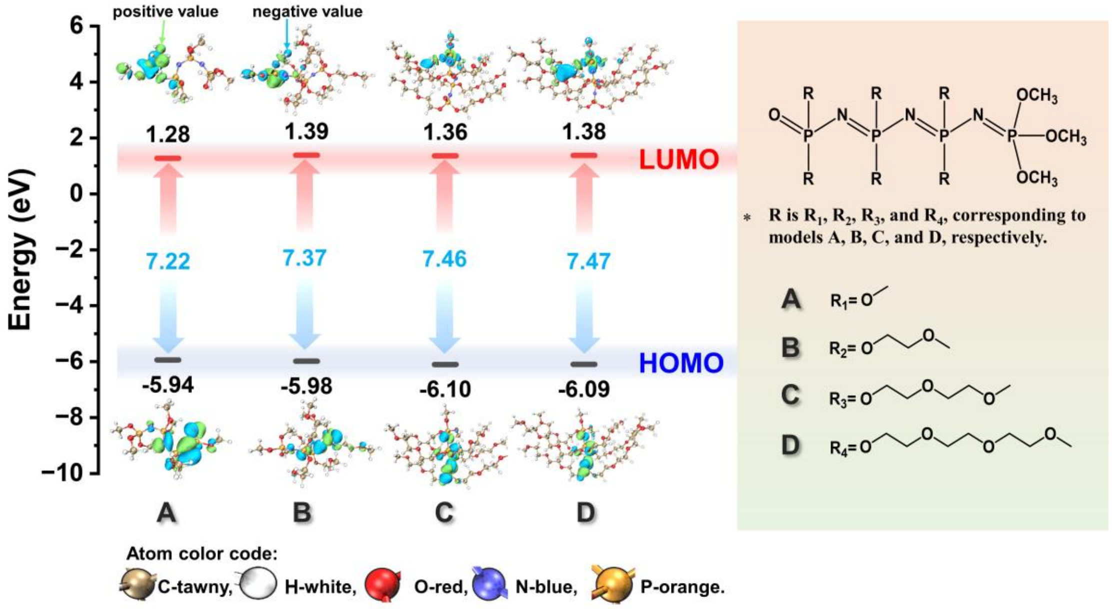

Supplementary Figure 6. Change in oxidation, reduction potentials and electrochemical stability windows with polyphosphazene side chain structure. DFT calculations were performed on fragment chain models on the right. Pictures show the position of the highest occupied molecular orbital (HOMO) and the lowest unoccupied molecular orbital (LUMO) levels. The relevant data files can be found in Supplementary Data 2.

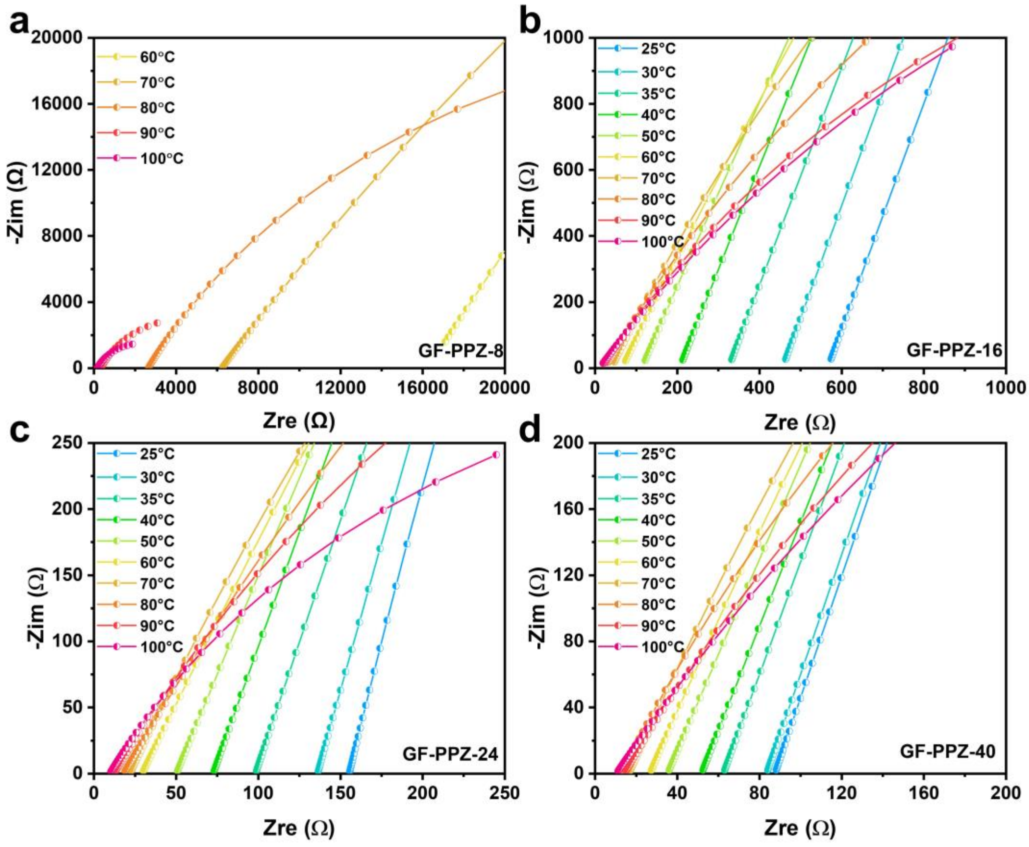

Supplementary Figure 7. Nyquist plots of the SS/LPEs/SS symmetrical cell at various temperatures with different LiTFSI contents: **a** GF-PPZ-8, **b** GF-PPZ-16, **c** GF-PPZ-24, **d** GF-PPZ-40.

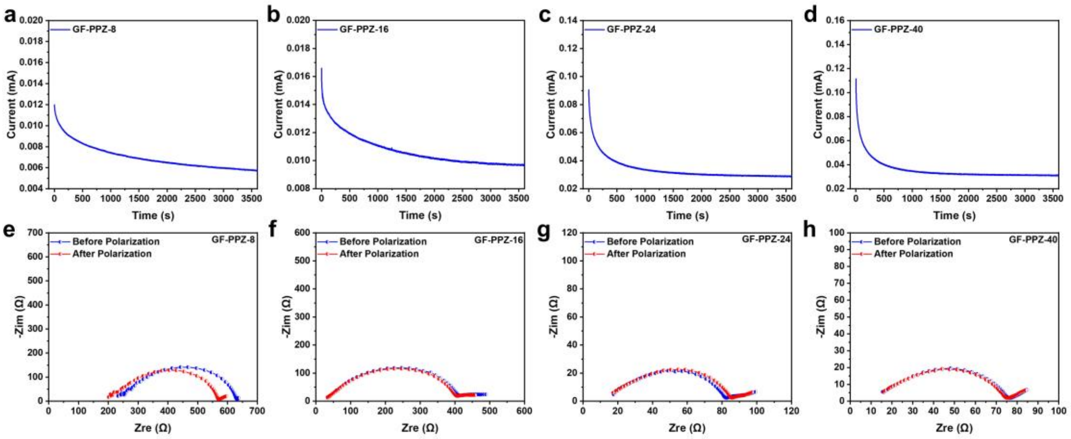

Supplementary Figure 8. The lithium-ion transference number test of LPEs at 90ºC. **a-d** Chronoamperometry profiles and **e-h** AC impedance spectra (before and after polarization) of GF-PPZ-8, GF-PPZ-16, GF-PPZ-24 and GF-PPZ-40, respectively.

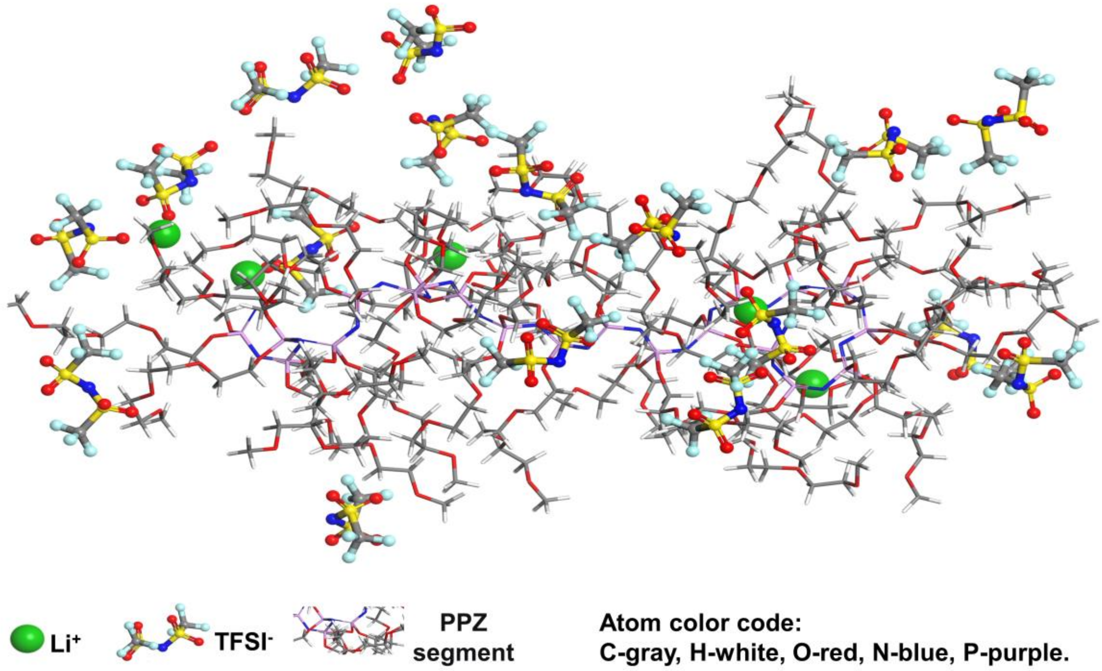

Supplementary Figure 9. Molecular dynamics (MD) simulation of the distribution of Li^+ and $TFSI^-$ in LPE (GF-PPZ-16). Molecular dynamics simulations data are provided as Supplementary Data 1 file.

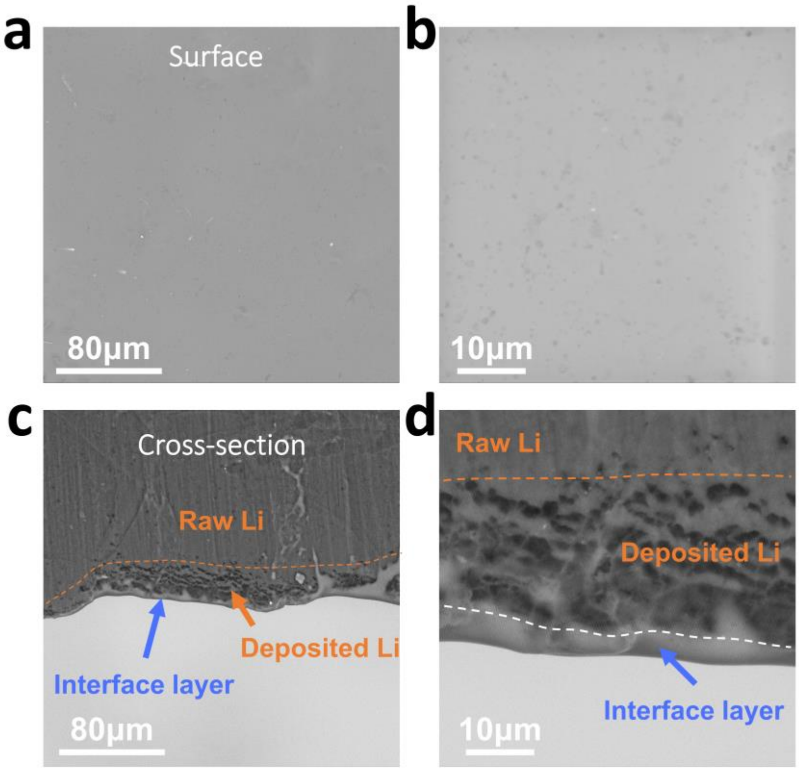

Supplementary Figure 10. Microscopic morphology of lithium metal electrode. SEM images of **a, b** surface and **c, d** cross-section of Li electrode in Li/GF-PPZ-16/Li cell after 2200 h operation.

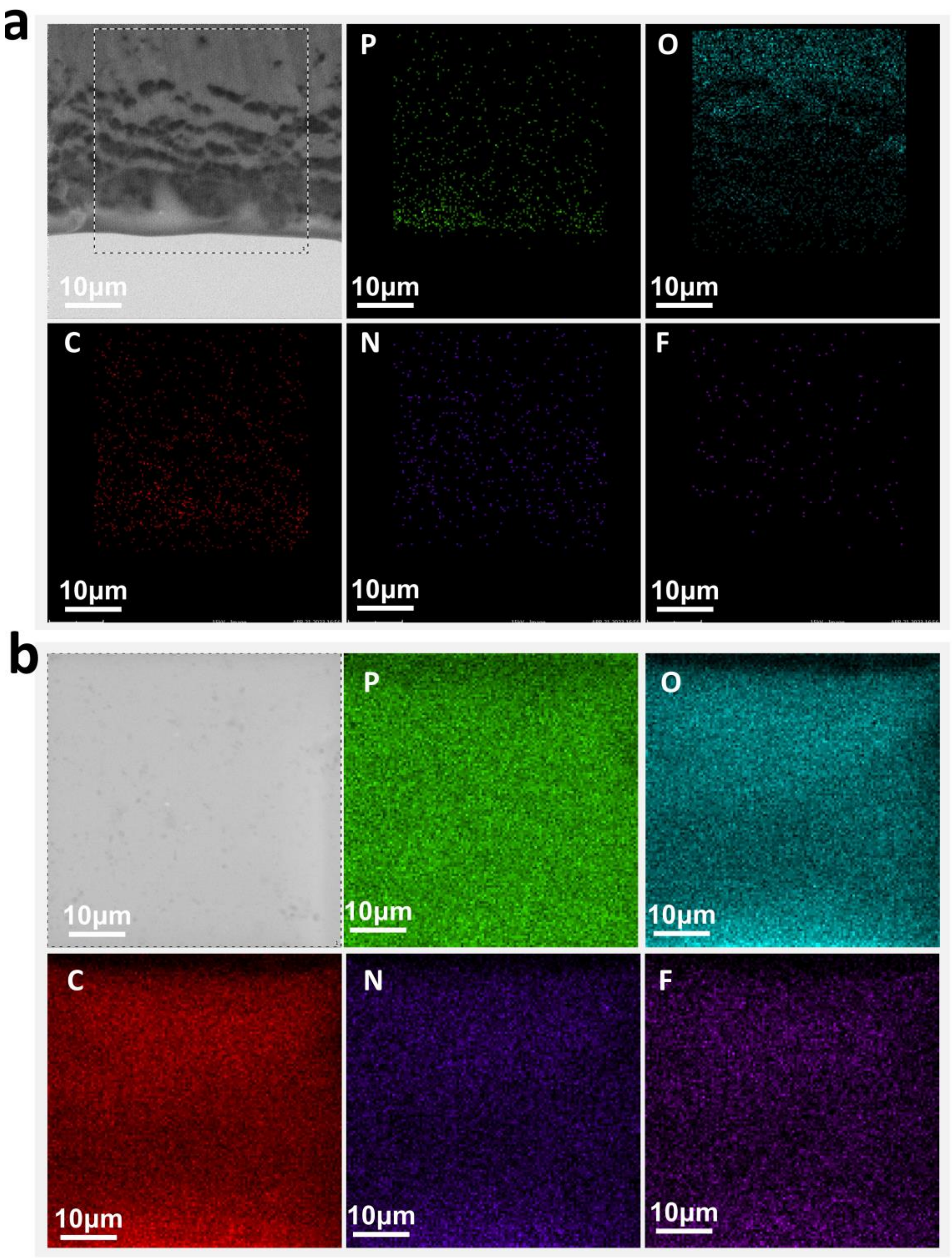

Supplementary Figure 11. Elemental distribution of lithium metal electrode. SEM and EDS mapping images of **a** cross-section and **b** surface of Li electrode in Li/GF-PPZ-16/Li cell after 2200 h operation.

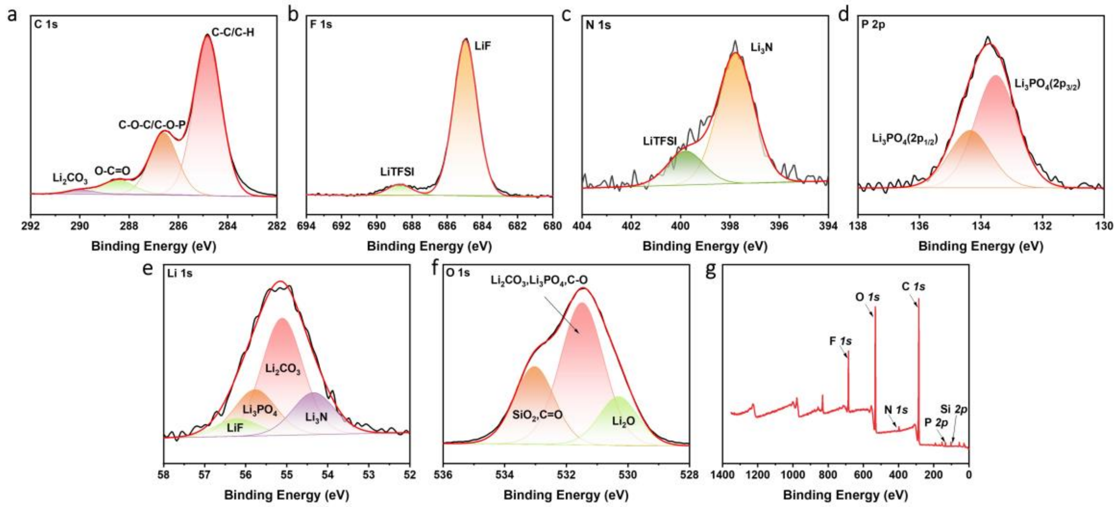

Supplementary Figure 12. XPS spectra of Li electrode surface in Li/GF-PPZ-16/Li cell after 2200 h operation: **a** C *1s*, **b** F *1s*, **c** N *1s*, **d** P *2p*, **e** Li *1s*, **f** O *1s*, **g** all elements.

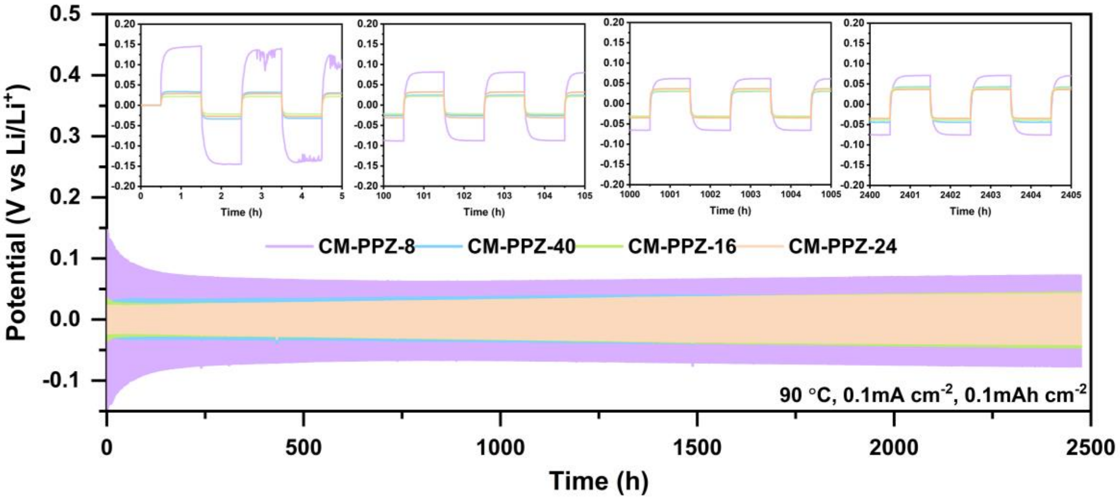

Supplementary Figure 13. Voltage profiles of lithium-metal plating/stripping in Li/LPEs/Li symmetrical cells with CM separator at 90 °C with a current density of 0.1 mA cm^{-2} and 0.1 mAh cm^{-2}. The insets are voltage profiles of Li/LPE/Li cells at 1-5 h, 100-105 h, 1000-1005 h and 2400-2405 h, respectively.

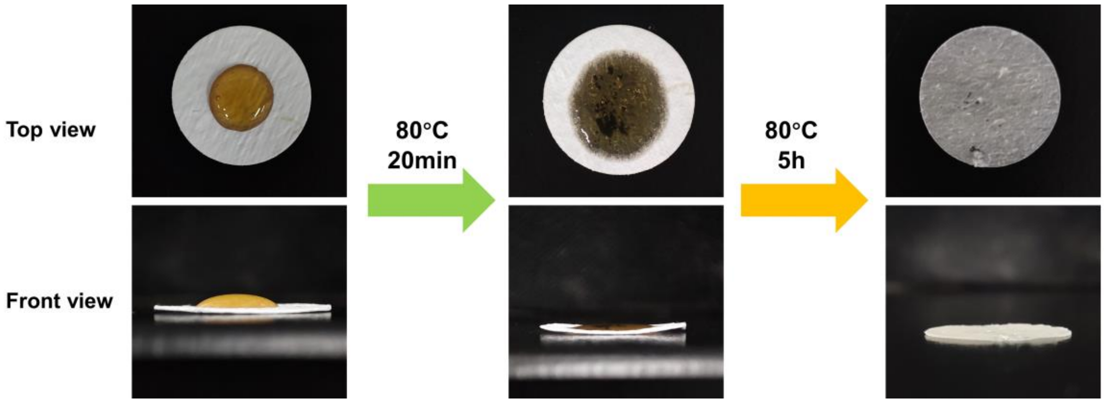

Supplementary Figure 14. Infiltration experiment of LPE (PPZ-40) to GF at 80 °C. No solvent was involved in this process.

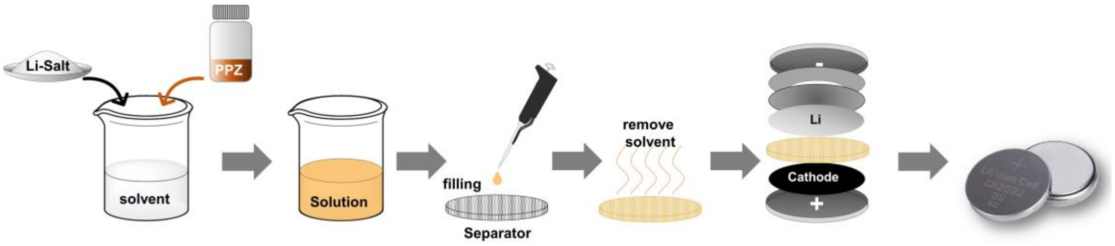

Supplementary Figure 15. Schematic diagram of loading LPEs onto a separator by the solution-assisted method.

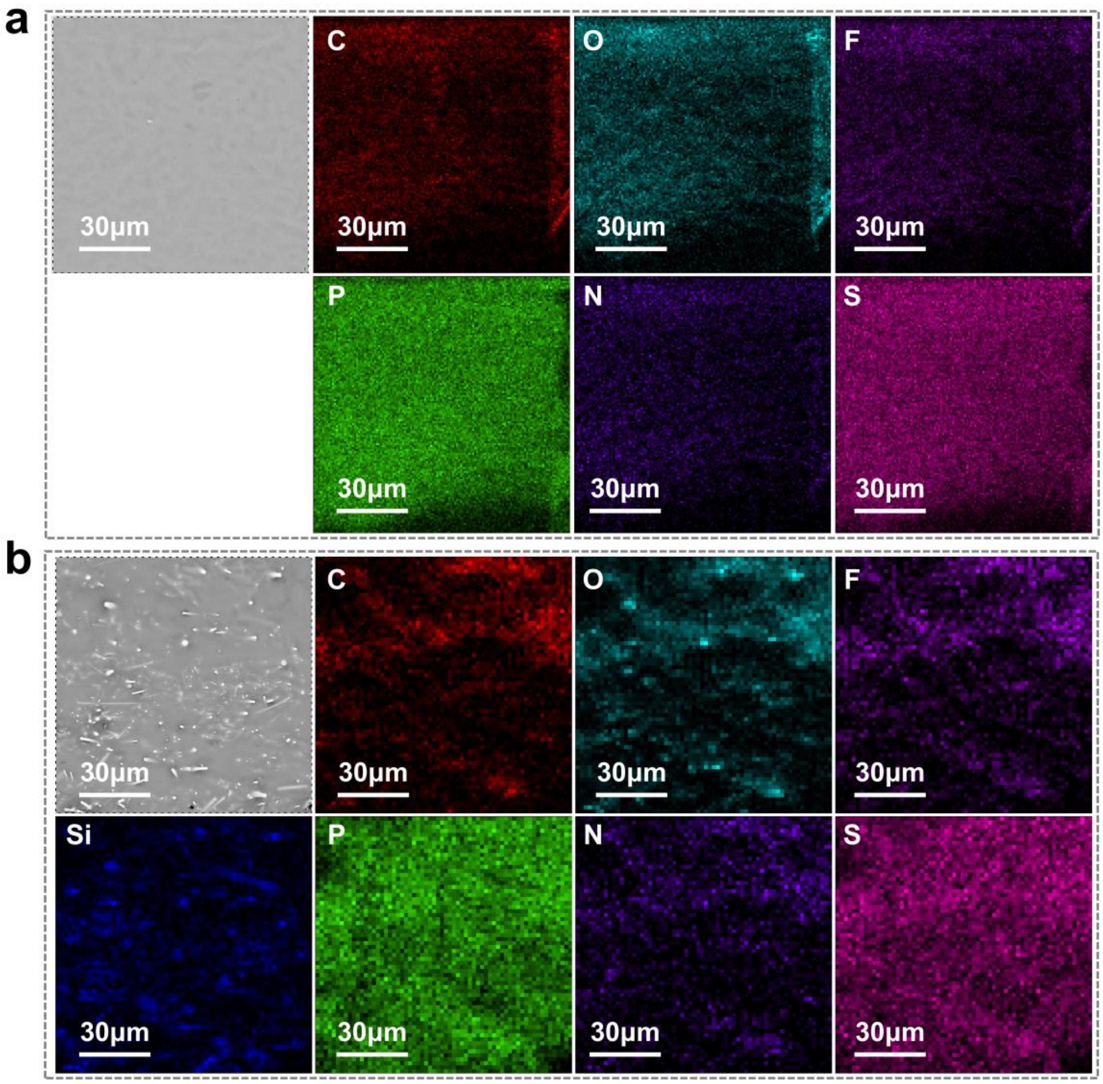

Supplementary Figure 16. Microscopic morphology and elemental distribution of electrolytes. SEM and EDS mapping images on **a** surface and **b** cross-section of GF-PPZ-16 electrolyte.

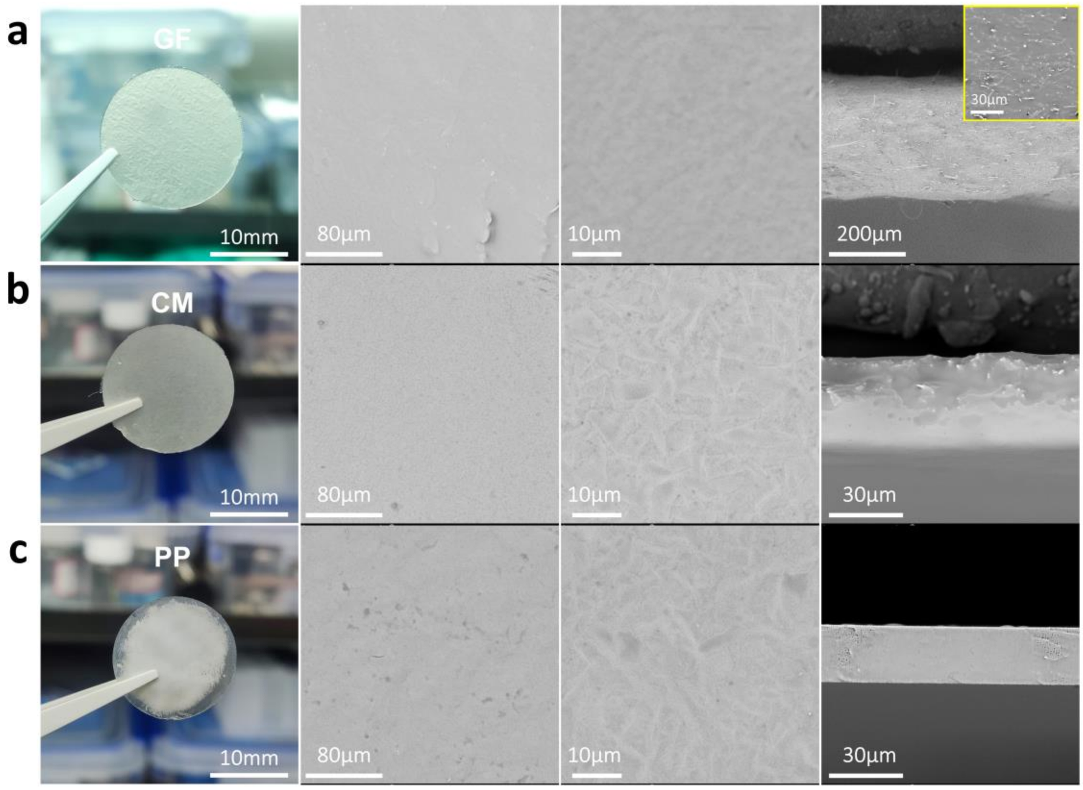

Supplementary Figure 17. The digital photographs and SEM images of the LPEs with different separators: **a** GF-PPZ-16, **b** CM-PPZ-16, and **c** PP-PPZ-16.

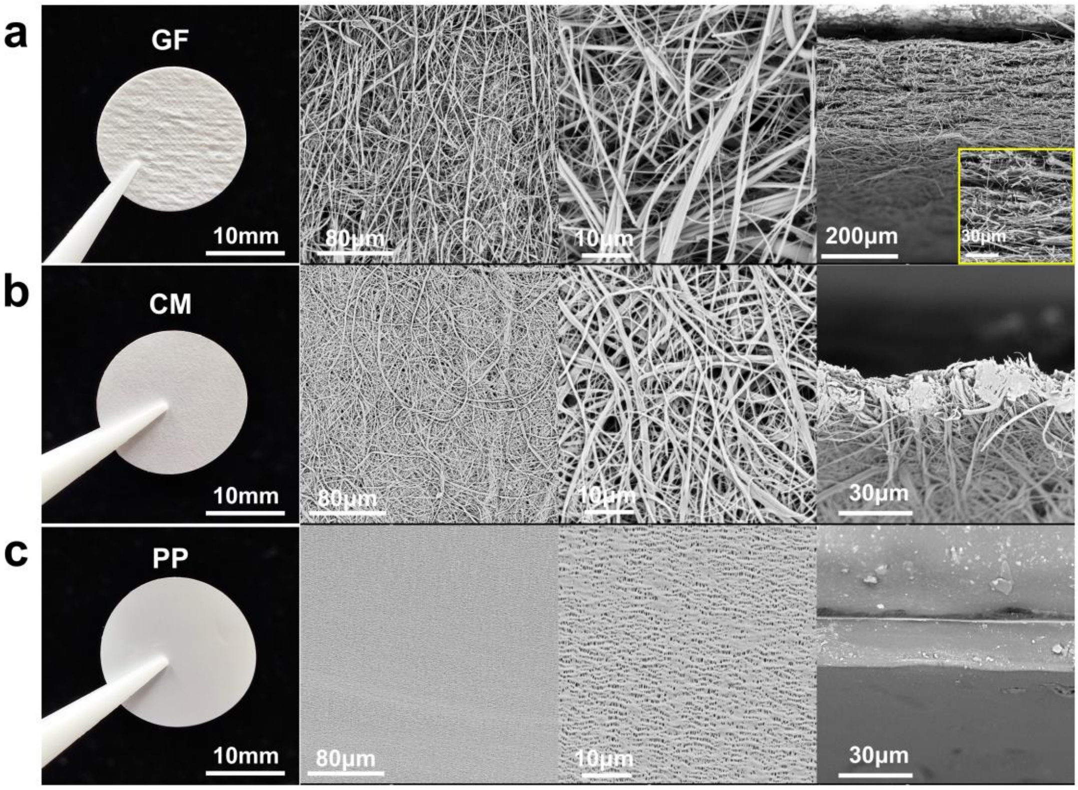

Supplementary Figure 18. The digital photographs and SEM images of different separator: **a** GF, **b** CM, and **c** PP.

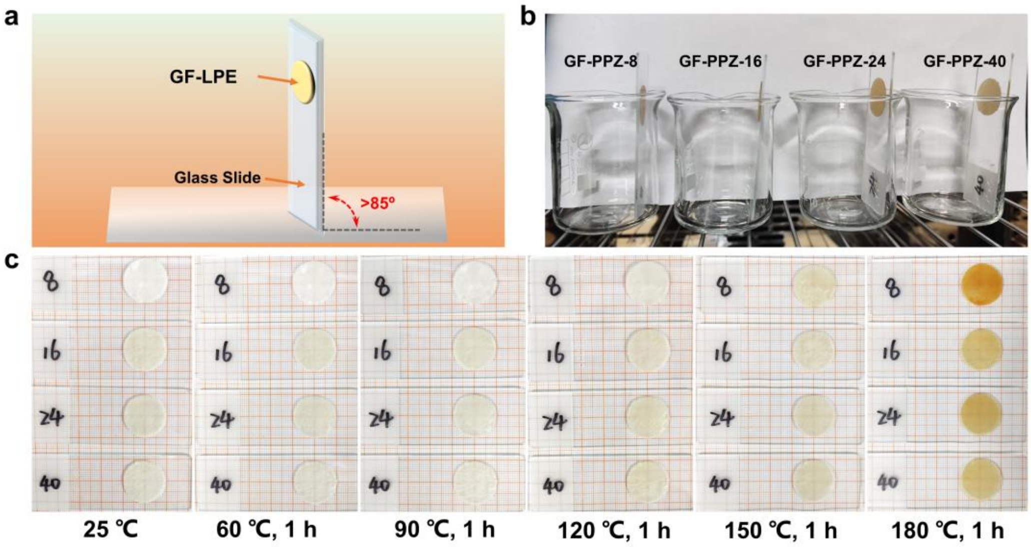

Supplementary Figure 19. Vertical heating test of GF-LPEs used to determine electrolyte leakage and thermal deformation. **a** Schematic diagram of vertical heating test, GF-LPE was adhered to the glass slide by its own adhesion. **b** Photos of the test process, samples were placed vertically in beakers and then heated in a blast oven. **c** Photos of samples heated at different temperatures for 1 h. Labels 8, 16, 24 and 40 correspond to GF-PPZ-8, GF-PPZ-16, GF-PPZ-24 and GF-PPZ-40, respectively. The high temperature air caused the color of the samples to darken.

As shown in Figure 19c, the GF-LPEs have good shape stability and there is no visible dimensional change, liquid spillage or leakage of the samples even after heating at 180 °C for 1 hour.

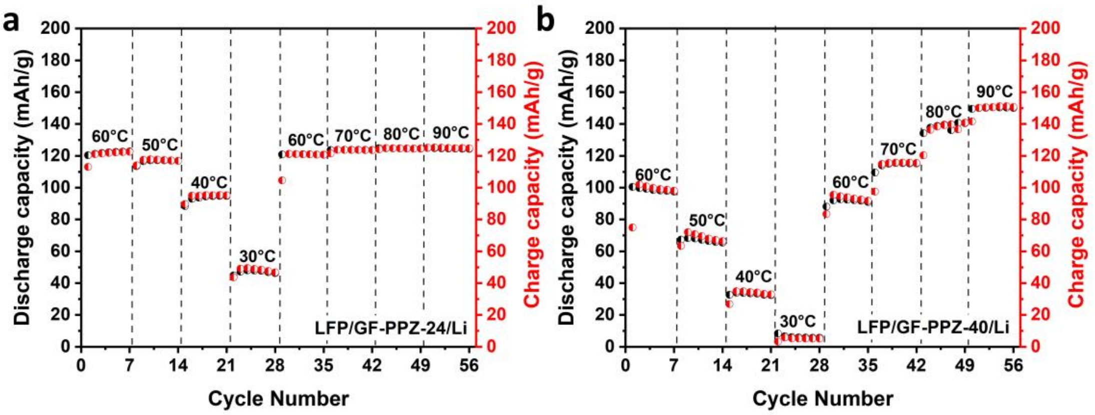

Supplementary Figure 20. Capacity performance of LFP/LPEs/Li cells at 0.5C with various temperatures for **a** GF-PPZ-24 and **b** GF-PPZ-40.

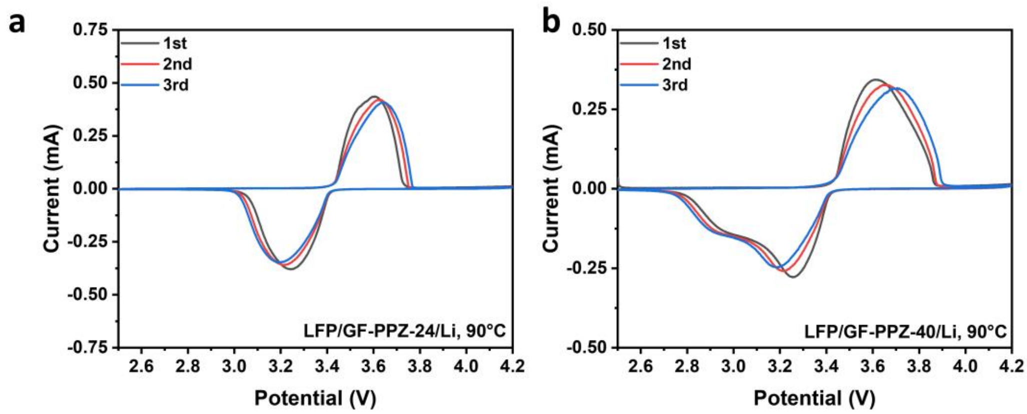

Supplementary Figure 21. The CV curves of **a** GF-PPZ-24 and **b** GF-PPZ-40 at 90 °C with a sweep rate of 0.1 mV s^{-1}.

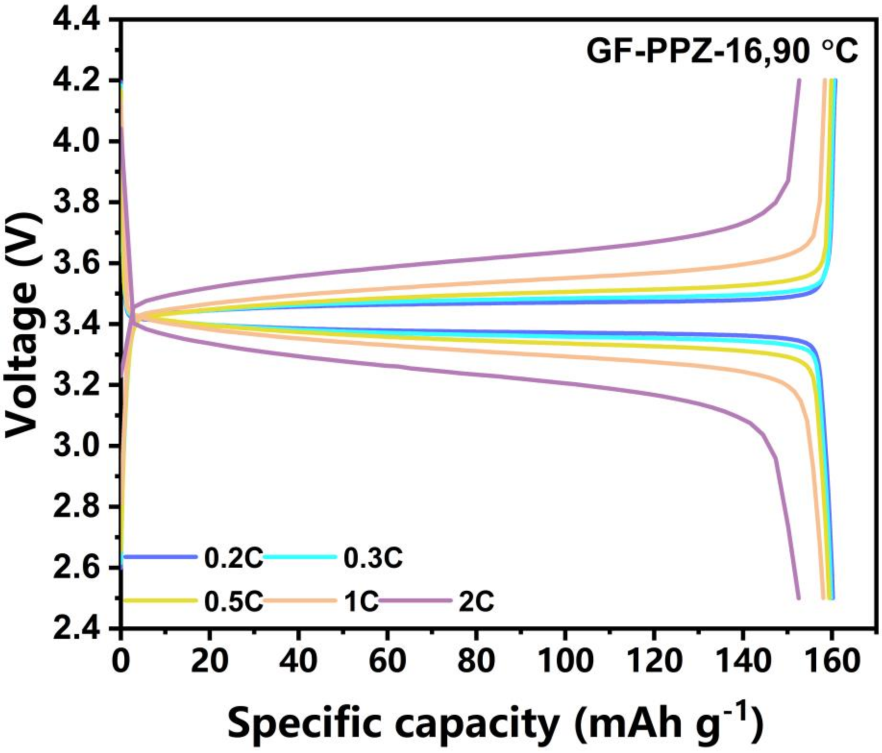

Supplementary Figure 22. The specific capacity-voltage curves of LFP/GF-PPZ-16/Li cell at 90 °C with different current densities.

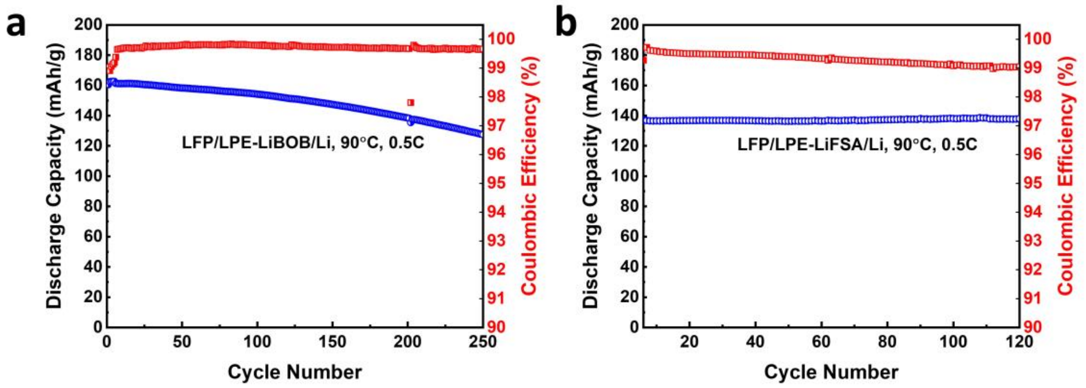

Supplementary Figure 23. Cycling performance of LPEs with different lithium salt: **a** LiBOB and **b** LiFSA. (O:Li=16:1).

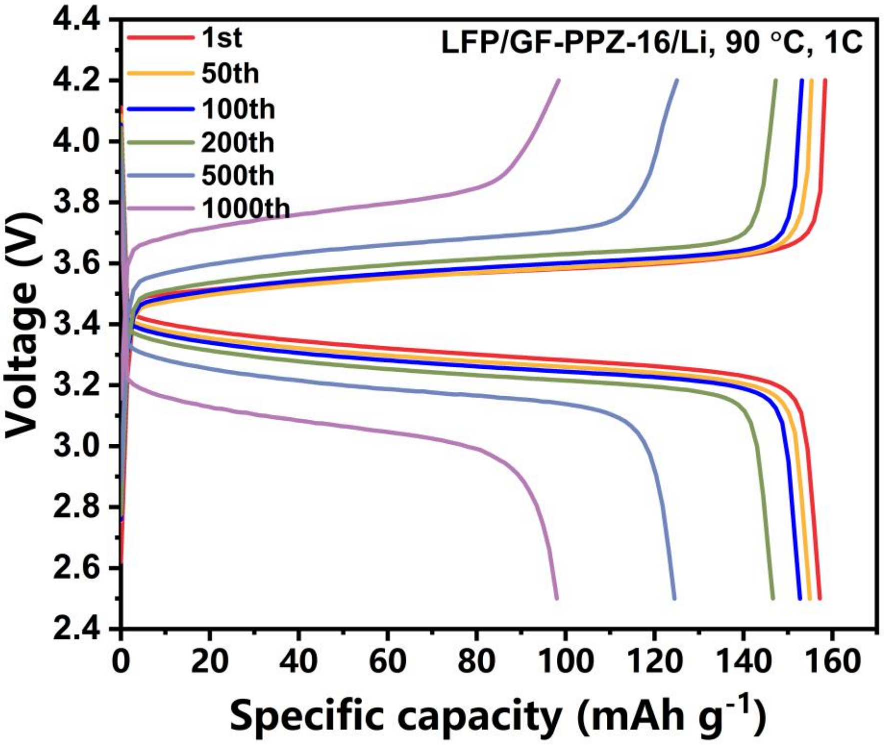

Supplementary Figure 24. The specific capacity-voltage curves of LFP/GF-PPZ-16/Li cell at 1C and 90 °C.

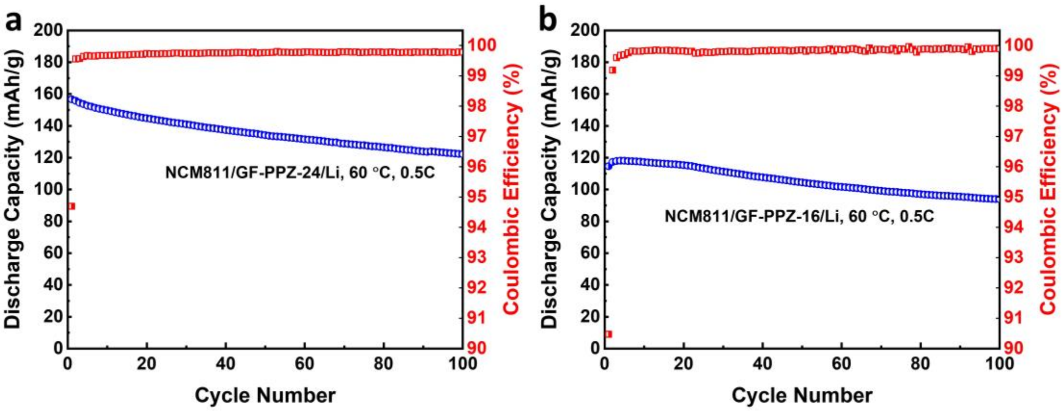

Supplementary Figure 25. The cycle performances of NCM811//Li cells with **a** GF-PPZ-24 and **b** GF-PPZ-16 electrolytes. The cells operated at 60 °C with a current density of 0.5C.

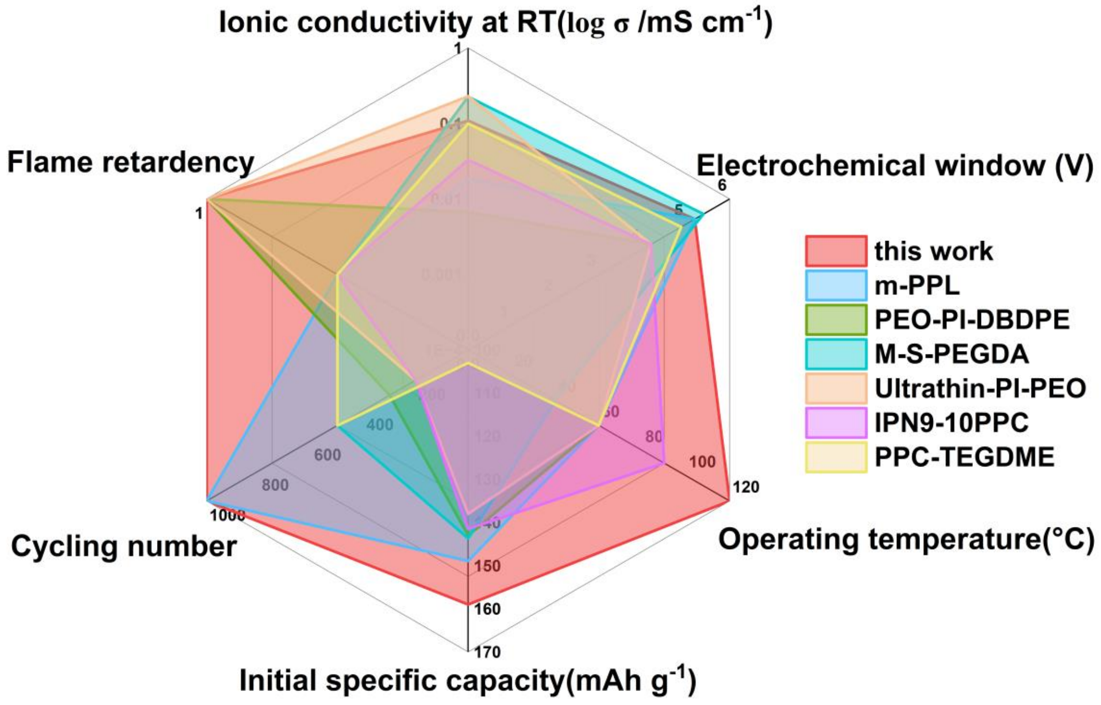

Supplementary Figure 26. Radar plots of the comprehensive performance comparison, and corresponding references can be found in Supplementary Table 6.

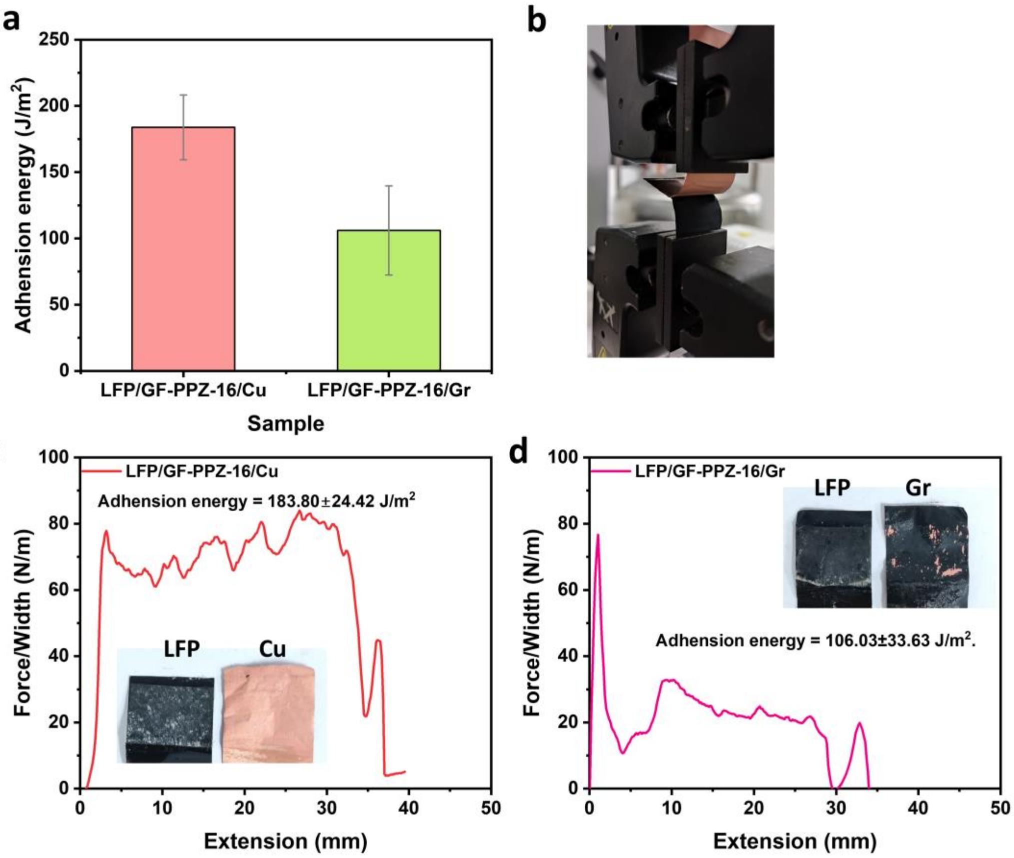

Supplementary Figure 27. Adhesion test of GF-PPZ-16 LPE to two battery structure. **a** Histogram with error bar for adhesion energy, bars denote S.D. **b** The optical image of the test. Adhesion test data of **c** LFP/GF-PPZ-16/Cu cell and **d** LFP/GF-PPZ-16/Gr cell, the insets show the electrodes after adhesion test. The adhesion of LPE to electrodes was higher than the structural strength of the self-made graphite anode, resulting in the graphite electrode being stripped from the copper foil during the test.

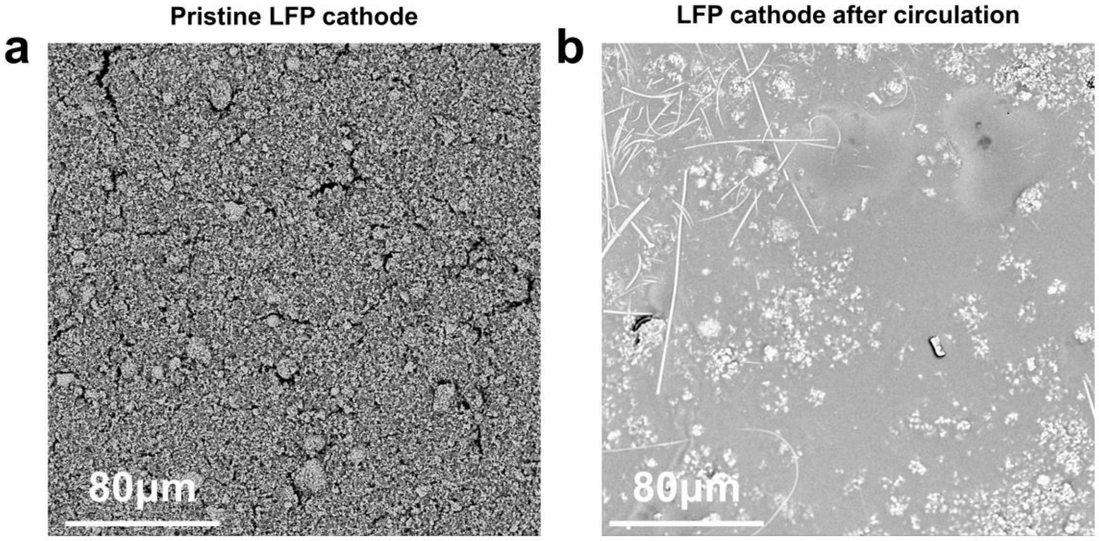

Supplementary Figure 28. SEM images of the surface of LFP cathode: **a** pristine and **b** after circulation. The cathode consists of LFP: PVDF: Super P=8:1:1.

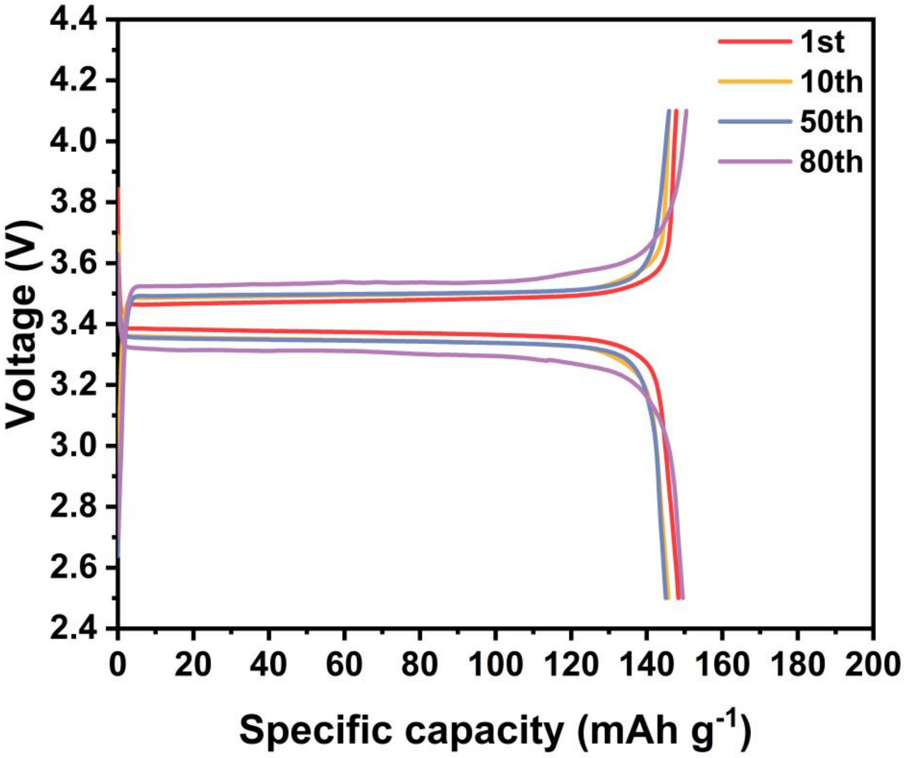

Supplementary Figure 29. The specific capacity-voltage curves of LFP/LPE/Li cell at 0.2C and 90 °C. Here, the electrolyte is CM-PPZ-16.

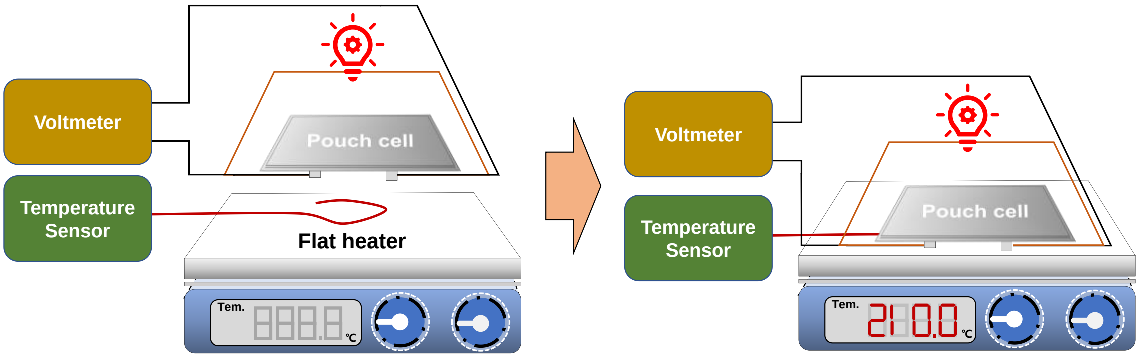

Supplementary Figure 30. Schematic diagram of the thermal abuse experiment device. A flat heater was used as the heat source, and the pouch cell was monitored by a tightly fitted temperature sensor. The voltmeter was directly connected to the positive and negative poles of the battery to detect real-time changes in the voltage of the small bulb.

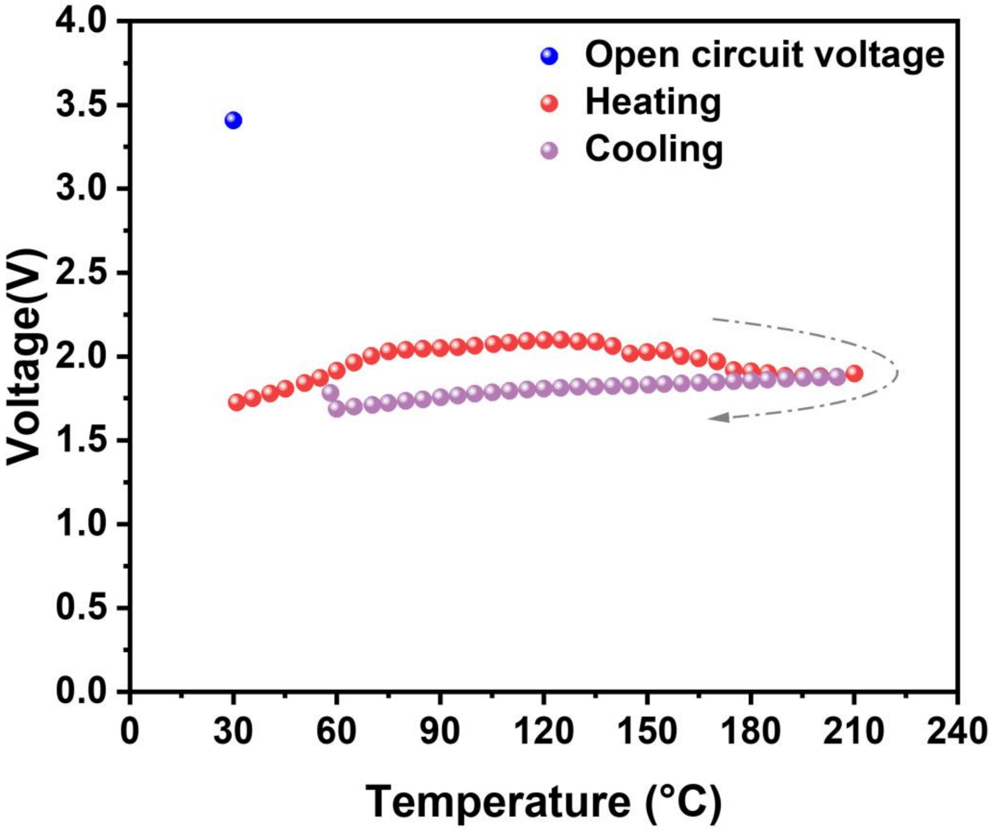

Supplementary Figure 31. Variation of the operating voltage of the pouch cell in thermal abuse test.

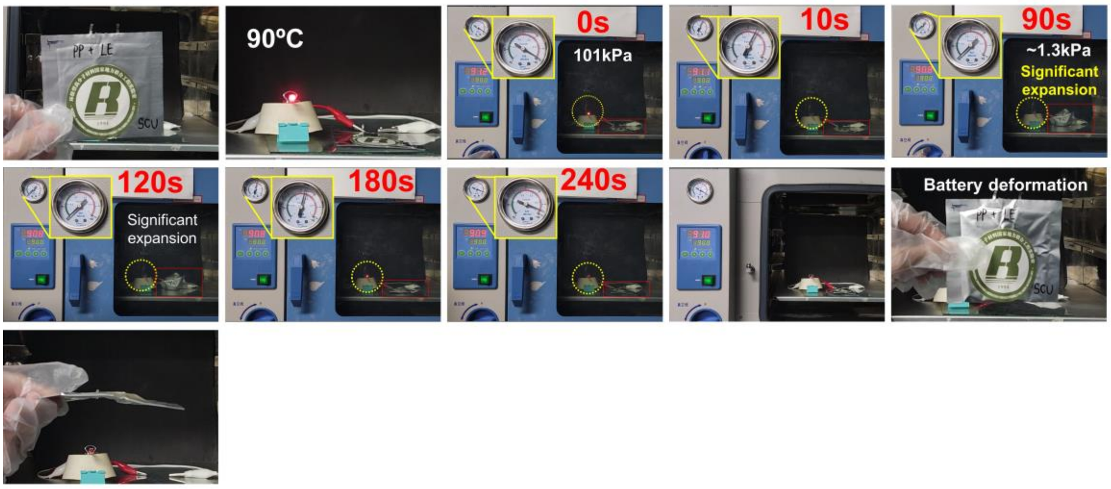

Supplementary Figure 32. The vacuum-heating test of LE-based pouch cell. The LFP/CM-LE/Li pouch cell run at 90 °C and ~1.3 kPa.

Significant expansion (Red wireframe) and failure (LED bulb goes off in yellow circle) were observed at low vacuum. The battery after the test was significantly deformed.

Supplementary Table 1. The proportion of $TFSI^-$ in three states in LPEs of different lithium concentrations.

$TFSI^-$ state	LiTFSI	PPZ-8	PPZ-16	PPZ-24	PPZ-40
AGG	100%	7.74%	0.43%	2.95%	0
CIP	0	79.95%	40.99%	18.99%	0
Free	0	12.31%	58.58%	78.06%	100%

AGG: aggregates, one $TFSI^-$ interacting with two or more Li^+.
CIP: contact ion pairs, one $TFSI^-$ interacting with a single Li^+.
Free: free $TFSI^-$ anion.

Supplementary Table 2. The glass transition temperature (T_g) of LPEs at a heating rate of 5 °C min^{-1}.

Sample	PPZ	PPZ-8*	PPZ-16*	PPZ-24*	PPZ-40*
T_g (°C)	-78.19	-31.49	-39.66	-53.60	-62.65

Supplementary Table 2. (continued)

Sample	GF	PPZ-16*	GF-PPZ-16*
T_g (°C)	-	-39.66	-38.34

* The number represents the molar ratio of the O atom to Li^+ ion in LPEs.

Supplementary Table 3. The decomposition data of LPEs in nitrogen at a heating rate of 10 °C min^{-1}.

Sample	$T_{5\%}$ (°C)	T_{max} (°C)	MDR* (% min^{-1})	Residue at 700 °C (wt%)
GF	-	-	-	97.46
LiTFSI	374.10	448.00	-12.65	2.60
PPZ	280.00	297.90/496.60	-30.74/-1.53	12.80
PPZ-16	230.00	236.20/376.00	-17.3/-4.87	12.21
GF-PPZ-16	230.50	243.60/395.10	-15.85/-4.08	22.36

*MDR=Maximum decomposition rate.

Supplementary Table 4. Ionic conductivity of LPEs with different LiTFSI contents at various temperatures, and activation energy (E_a) values were calculated by Vogel–Tamman–Fulcher (VTF) empirical equation.

Temperature	Ionic conductivity (σ, S cm^{-1})			
(°C)	GF-PPZ-8	GF-PPZ-16	GF-PPZ-24	GF-PPZ-40
25	-	3.60E-05	7.78E-05	1.09E-04
30		4.48E-05	8.88E-05	1.14E-04
35		6.29E-05	1.23E-04	1.53E-04
40	-	9.93E-05	1.67E-04	1.84E-04
50	-	1.80E-04	2.41E-04	2.70E-04
60	8.69E-06	3.12E-04	4.13E-04	3.58E-04
70	2.17E-05	5.14E-04	5.50E-04	5.78E-04
80	4.38E-05	8.70E-04	6.75E-04	7.47E-04
90	8.28E-05	1.34E-03	9.79E-04	8.64E-04
100	1.58E-04	2.01E-03	1.27E-03	9.30E-04
VTF-E_a (kJ/mol)	26.11	11.36	8.56	8.06

Note: The ultrahigh concentration of lithium salt in PPZ-8 caused that the ionic conductivity below 60 °C could not been accurately measured.

Supplementary Table 5. The lithium-ion transference number (t_{Li^+}) and effective Li^+ conductivity (σ_{Li^+}) of LPEs with different LiTFSI contents at various temperatures. (σ_{Li^+}, S cm^{-1})

Temperature (°C)	GF-PPZ-8		GF-PPZ-16		GF-PPZ-24		GF-PPZ-40	
	t_{Li}^+	σ_{Li}^+	t_{Li}^+	σ_{Li}^+	t_{Li}^+	σ_{Li}^+	t_{Li}^+	σ_{Li}^+
25	-	-	0.37	1.33E-05	0.16	1.24E-05	0.11	1.20E-05
30	-	-	0.27	1.21E-05	0.20	1.78E-05	0.09	1.03E-05
40	-	-	0.28	2.78E-05	0.16	2.67E-05	0.09	1.66E-05
50	-	-	0.30	5.40E-05	0.17	4.10E-05	0.09	2.43E-05
60	0.46	4.00E-06	0.31	9.67E-05	0.16	6.61E-05	0.10	3.58E-05
70	0.44	9.55E-06	0.34	1.75E-04	0.16	8.80E-05	0.12	6.94E-05
80	0.39	1.71E-05	0.32	2.78E-04	0.15	1.01E-04	0.10	7.47E-05
90	0.38	3.15E-05	0.30	4.02E-04	0.16	1.57E-04	0.10	8.64E-05

Supplementary Table 6. The comprehensive performance of LPEs and recently reported advanced LEs and SPEs for LMBs.

Type	Electrolyte	σ at RT ($mS\ cm^{-1}$)	ESW (V)	Cell performance①	Operating Temperature (°C)	Flame retardancy②
**LE**	**EGDBE-E[5]**	4.01	N/A	（NCM811）~200 mAh g^{-1}, 200 cycles at 0.5C, CLR≈0.1%③	60	0
	**Pyrolux™-LiBOB/EC/PC/VC[6]**	3.38	5.0	155 mAh g^{-1}, 35 cycles at 0.33C, CLR<0.29%	120	1
	**ADFN[7]**	N/A	4.65	~160 mAh g^{-1}, 100 cycles at 1C	90	0
**SPE**	**m-PPL[8]**	0.0185	5.2	148.9 mAh g^{-1}, 1000 cycles at 1C, CLR=0.024%	60	0
	**M-S-PEGDA[9]**	0.226	5.4	143.7 mAh g^{-1}, 500 cycles at 0.5C, CLR=0.029%	40	0
	**PPC-TEGDME[10]**	0.89(60°C)	4.89	103 mAh g^{-1}, 500 cycles at 1C, CLR=0.046%	60	0
	**PEO-PI-DBDPE[11]**	0.0067	4.2	143 mAh g^{-1}, 300 cycles at 0.5C, CLR=0.03%	60	1
	**IPN9-10PPC[12]**	0.033 (40°C)	4.2	141.5 mAh g^{-1}, 200 cycles at 0.2C, CLR=0.037%	90	0
	**Ultrathin-PI-PEO[13]**	0.23(30°C)	4.2	138 mAh g^{-1}, 200 cycles at 0.5C.	60	0
**LPE**	**This work**	0.109	5.2	160.4 mAh g^{-1}, 1000 cycles at 0.5C, CLR=0.036%	60	1
				159 mAh g^{-1}, 1000 cycles at 1C, CLR=0.038%	90	1
				160 mAh g^{-1}, 100 cycles at 2C, CLR=0.066%	120	1

① Unmarked cathode defaults to LFP, and unmarked anode defaults to Li anode.
② 0 represents flammable, and 1 represents nonflammable.
③ CLR=Capacity loss rate (per cycle).

Supplementary References

1. Cabañero Martínez MA, *et al.* Are Polymer‐Based Electrolytes Ready for High‐Voltage Lithium Battery Applications? An Overview of Degradation Mechanisms and Battery Performance. *Adv Energy Mater* **12**, 2201264 (2022).
2. Li Z, Zhao Y, Tenhaeff WE. Determining the Absolute Anodic Stability Threshold of Polymer Electrolytes: A Capacity-Based Electrochemical Method. *Chem Mater* **33**, 1927-1934 (2021).
3. Yongyao Xia TF, Kuniaki Tatsumi, Pier Paolo Prosini, Tetsuo Sakai. Thermal and electrochemical stability of cathode materials in solid polymer electrolyte. *J Power Sources* **92**, 234-243 (2001).
4. Homann G, Stolz L, Nair J, Laskovic IC, Winter M, Kasnatscheew J. Poly(Ethylene Oxide)-based Electrolyte for Solid-State-Lithium-Batteries with High Voltage Positive Electrodes: Evaluating the Role of Electrolyte Oxidation in Rapid Cell Failure. *Sci Rep* **10**, 4390 (2020).
5. Wang Z, Chen C, Wang D, Zhu Y, Zhang B. Stabilizing Interfaces in High-Temperature NCM811-Li Batteries via Tuning Terminal Alkyl Chains of Ether Solvents. *Angew Chem Int Ed Engl* **62**, e202303950 (2023).
6. Kohlmeyer RR, *et al.* Pushing the thermal limits of Li-ion batteries. *Nano Energy* **64**, 103927 (2019).
7. Zheng T, *et al.* Cocktail therapy towards high temperature/high voltage lithium metal battery via solvation sheath structure tuning. *Energy Storage Mater* **38**, 599-608 (2021).
8. Wang Z, Shen L, Deng S, Cui P, Yao X. 10 mum-Thick High-Strength Solid Polymer Electrolytes with Excellent Interface Compatibility for Flexible All-Solid-State Lithium-Metal Batteries. *Adv Mater* **33**, 2100353 (2021).
9. Wang H, *et al.* Thiol-Branched Solid Polymer Electrolyte Featuring High Strength, Toughness, and Lithium Ionic Conductivity for Lithium-Metal Batteries. *Adv Mater* **32**, e2001259 (2020).
10. Didwal PN, Verma R, Nguyen AG, Ramasamy HV, Lee GH, Park CJ. Improving Cyclability of All-Solid-State Batteries via Stabilized Electrolyte-Electrode Interface with Additive in Poly(propylene carbonate) Based Solid Electrolyte. *Adv Sci* **9**, 2105448 (2022).
11. Cui Y, Wan JY, Ye YS, Liu K, Chou LY. A Fireproof, Lightweight, Polymer-Polymer Solid-State Electrolyte for Safe Lithium Batteries. *Nano Lett* **20**, 1686-1692 (2020).
12. Zheng Y, Li X, Li CY. A novel de-coupling solid polymer electrolyte via semi-interpenetrating network for lithium metal battery. *Energy Storage Mater* **29**, 42-51 (2020).
13. Wan J, *et al.* Ultrathin, flexible, solid polymer composite electrolyte enabled with aligned nanoporous host for lithium batteries. *Nat Nanotechnol* **14**, 705-711 (2019).
