# Supplementary material for: Non-flammable solvent-free liquid polymer electrolyte for lithium metal batteries
Source: Nat Commun. 2023 Aug 1;14:4617. doi: 10.1038/s41467-023-40394-8 (PMC10394022; doi:10.1038/s41467-023-40394-8)
Supplement: Supplementary file 3 — Description of Additional Supplementary Files [file 41467_2023_40394_MOESM3_ESM.pdf]

Description of Additional Supplementary Files

Supplementary Data 1: Molecular dynamics simulation data of PPZ-16 electrolyte. File NVT-1ps is the atomic coordinates at 1ps, and File NVT-2000ps is the atomic coordinates at 2000 ps.

Supplementary Data 2: Density functional theory calculations data for models of polyphosphazene fragments with different side chains. Files A, B, C, and D are the optimized atomic coordinates of models A, B, C and D in Supplementary Figure 6, respectively. The side chains of models A, B, C and D are methoxy, methoxyethoxy, methoxydiethoxy and methoxytriethoxy, respectively.
